# Characterization of proteins regulated by interleukin-4 in 3T3-L1 adipocytes

**DOI:** 10.1186/s40064-015-0980-0

**Published:** 2015-06-04

**Authors:** Ming-Yuh Shiau, Hsu-Feng Lu, Yih-Hsin Chang, Yen-Chih Chiu, Yung-Luen Shih

**Affiliations:** Department of Nursing, College of Medicine & Nursing, Hungkuang University, Taichung, Taiwan; Department of Clinical Pathology, Cheng Hsin General Hospital, Taipei, Taiwan; Department of Biotechnology and Laboratory Science in Medicine, National Yang-Ming University, Taipei, Taiwan; Department of Pathology and Laboratory Medicine, Shin Kong Wu Ho-Su Memorial Hospital, Taipei, Taiwan; School of Medical Laboratory Science and Biotechnology, Taipei Medical University, Taipei, Taiwan; School of Medicine, Fu-Jen Catholic University, New Taipei City, Taiwan

**Keywords:** Interleukin-4, Adipocytes, Metabolism, Proteomics

## Abstract

Obesity is closely associated with metabolic syndrome, type 2 diabetes mellitus (T2DM) and cardiovascular diseases. Our previous reports uncover the significant associations between *interleukin-4 (IL-4)/IL-4 receptor* genotypes and T2DM, as well as *IL-4* genotypes and high density lipoprotein-cholesterol. Theses observations suggest that IL-4 harbors the capacity to regulate lipid metabolism. The present study is aimed at further elucidating regulatory roles of IL-4 to lipid metabolism by identifying putative proteins in 3T3-L1 adipocytes which are differentially expressed under IL-4 treatment. Proteins in mature 3T3-L1 adipocytes with altered expression levels under IL-4 treatment were identified by proteomic strategy. Our results revealed that IL-4 up-regulated levels of ATP synthase δ chain, Cytochrome *c* reductase, Pyrophsphatase and Vimentin, whereas, Alpha-enolase, Gelsolin, Vinculin and Valosin were down-regulated. These observations suggest that IL-4 promotes energy metabolism and inhibit lipid deposits in adipocytes by up-regulating proteins accelerating ATP synthesis. Our results suggest that IL-4 facilitates adipocytes metabolism to catabolism with a favorable condition for lipolysis. These catabolized lipids in adipocytes triggered by IL-4 might either be released into periphery or metabolized intracellularlly, and modulate systemic energy metabolism.

## Background

Obesity is characterized by excess accumulation of lipids in intra-abdominal adipose tissue. The enlargement of adipose tissue is a combination of increased cell number (hyperplasia), size (hypertrophy) and formation (adipogenesis) of adipocytes (Cornelius et al. 1994; Sorisky 1999). Obesity has become an epidemic problem globally, affecting persons of all ages in both developed and developing countries. In 2010, the World Health Organization (WHO) estimated that about 2.1 billion adults were overweight (body Mass Index [BMI] > 25), and at least 400 million of them were obese (BMI > 30) (World Health Organization 2014). Obesity is also the major risk factor leading to insulin resistance and type 2 diabetes mellitus (T2DM), with 44 % of the diabetic burden are attributable to overweight and obesity. Moreover, overweight and obesity are leading risks for global deaths, causing death toll of 3.4 million adults each year. Therefore, obesity has become an epidemic health problem due to its global increasing prevalence and its close association with multiple metabolic abnormalities.

Adipose tissue is an important endocrine organ that secretes a variety of biologically active molecules (adipokines) (Bradley et al. 2005; Trayhurn and Beattie 2001). In addition to regulating energy metabolism, these adipokines also take part in immune responses and cardiovascular tone. Dysregulated production of these adipokines is implicated in obesity and related metabolic consequences (Chaldakov et al. 2003; Lyon et al. 2003). IL-6, one of the type 2 T helper cell (Th2) cytokines with the activity to specifically regulate Th1/Th2 balance, is elevated in T2DM subjects (Paul and Seder 1994). Accordingly, immune responses are suggested to play certain roles in obesity and the closely related metabolic abnormalities. In this context, differentially expressed proteins of adipocytes in response to external stimuli may regulate adipocyte behavior and contribute to metabolic abnormalities.

Interleukin-4 (IL-4) is another Th2 cytokine which mediates Th1/Th2 balance and immune responses by regulating the production of pro-inflammatory mediators from macrophages (Paul 1997; Garcia-Zepeda et al. 1996; Kang et al. 2008). Our previous reports identified the association between *IL-4/IL-4R* genotypes and T2DM, as well as between *IL-4* genotypes and high density lipoprotein-cholesterol (HDL-C) (Ho et al. 2010; Chang et al. 2012a). Our animal study reveals that IL-4 participates in lipid metabolism by inhibiting triglyceride accumulation in fat tissues, which leads to decreased weight gain and fat mass (Chang et al. 2012b). It suggests that IL-4 is involved in diabetic susceptibility and complications through its capacity of regulating insulin sensitivity, glucose tolerance and lipid metabolism. Our observations echo the hypothesis from Elbe-Burger *et al.* that IL-4 participates in lipid metabolism (Elbe-Burger et al. 2002). Our most recent report indicates that IL-4 harbors anti-lipogenic ability by suppressing adipocytes differentiation and promoting lipolysis in mature adipocytes (Tsao et al. 2014). The above results indicate that IL-4 regulates energy metabolism by promoting catabolism rather than energy storage through modulating adipocytes behaviors.

In this context, the aim of the present study is to identify the effects of IL-4 on protein expression profiles of adipocytes by proteomic strategy for further addressing the role of IL-4 in metabolism and metabolic pathogenesis. Our results suggest that IL-4 potentiates adipocytes metabolism to catabolism with a favorable condition for lipid decomposition. These catabolized lipids in adipocytes triggered by IL-4 might either be released into periphery or metabolized intracellularlly, and subsequently modulate systemic energy metabolism.

## Results and discussion

IL-4 has been suggested to participate in lipid metabolism by inducing peroxisome proliferator-activated receptor-γ expression in macrophages and monocytes (Ricote et al. 2000; Elbe-Burger et al. 2002). In support of the above hypothesis, our previous study reveals that IL-4 promotes lipolysis by boosting hormone sensitive lipase (HSL) activity and translocation in adipocytes. It indicates that the decrease of lipid deposits in adipocytes under IL-4 treatment results from the pro-lipolytic activity of IL-4 through modulating HSL activity to inhibit adipocytes differentiation and lipid accumulation (Tsao et al. 2014). For further addressing the roles of IL-4 in lipid metabolism, the present study aimed at characterizing proteins that are regulated by IL-4 in 3T3-L1 mature adipoctyes by proteomic techniques.

**Fig. 1:**
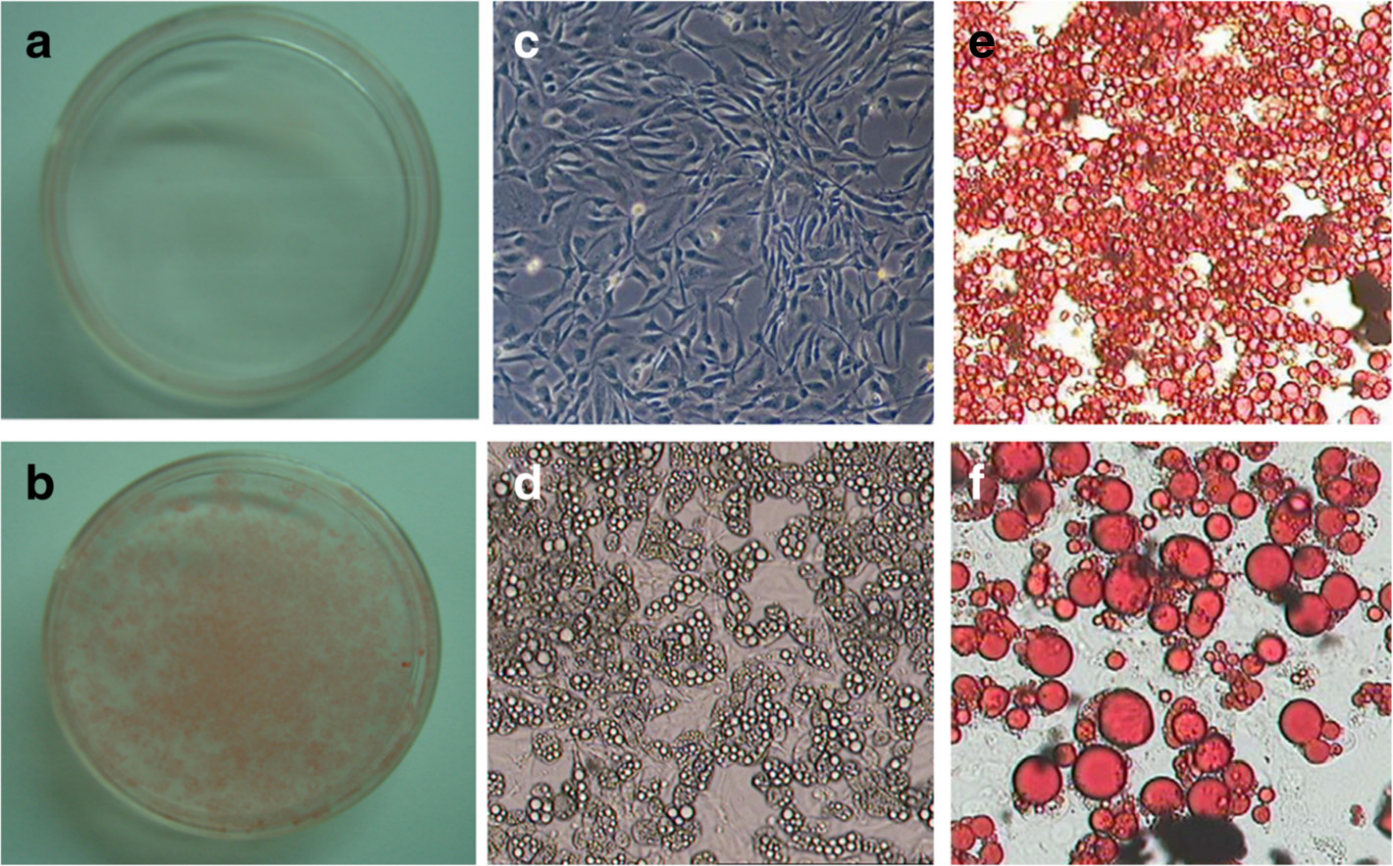
3T3-L1 pre-adipocytes were cultured for 8 days in the absence (**a**) or presence (**b**) of differentiation inducing agents, and subject to Oil-red O staining. The morphological characteristics of cells before and after differentiation were shown as (**c**) and (**d**), respectively; (**e**) and (**f**) showed the amplified Oil-red O stating results of (**b**)

**Fig. 2:**
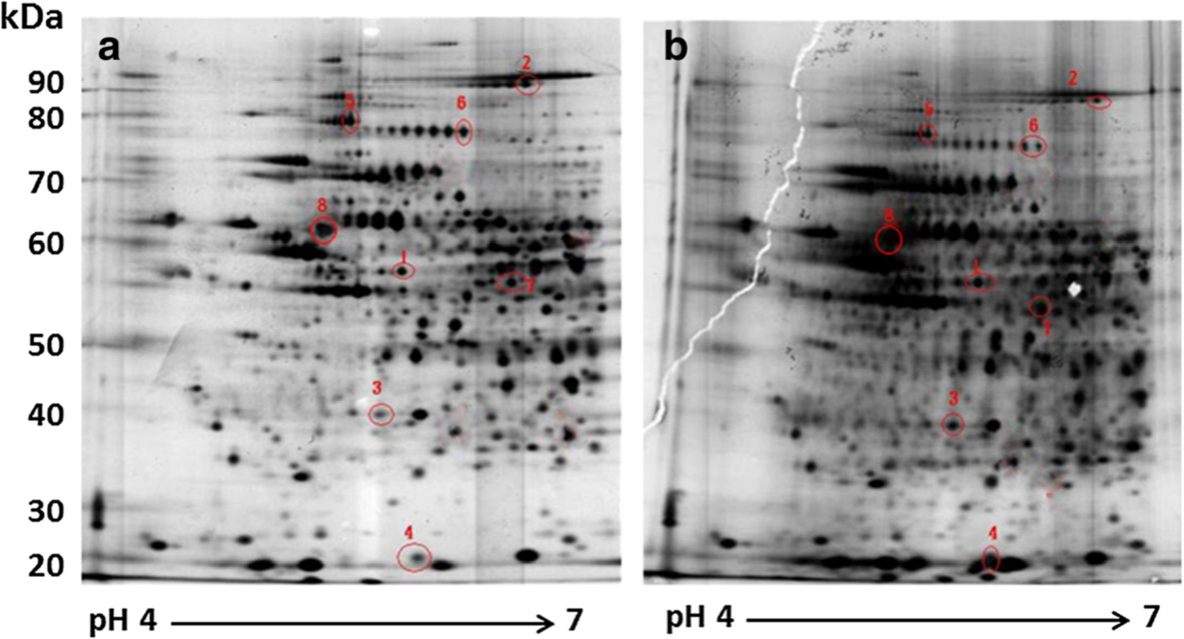
Representative 2-dimensional protein profiles of mature 3T3-L1 adipocytes. Proteins (120 μg) from fully differentiated 3T3-L1 adipocytes in the absence (**a**) or presence of IL-4 treatment (**b**; 50 *n*g/mL for 30 min) were separated by 2-dimensional electrophoresis (pH 4–7, 9-15 % SDS-PAGE) and visualized with silver stain

For achieving our study goal, the cell model system for 3T3-L1 adipocytes differentiation was firstly established as described (Tsao et al. 2014), and the extent of differentiation was evaluated by Oil-Red O (ORO) staining (Fig. 1). Dose response (10, 25 and 50 *n*g/mL) and time course (30 min to 24 h) experiments of IL-4 treatment in mature 3T3-L1 adipocytes were then examined to exclude the potential influences of protein expression patterns by cytotoxic effects induced by IL-4 for establishing optimal experimental condition. No apoptosis or necrosis of 3T3-L1 cells were observed under IL-4 treatment, and no significant difference in total amounts of proteins was observed (data not shown). Then proteomic analysis of putative immediate alterations in protein expression profiles in response to short term IL-4 treatment (30 min of 50 *n*g/mL IL-4) was analyzed. Figure 2 shows the representative 2-dimensional electrophoretic results of mature adipocytes protein expression profile in the absence (A) or presence (B) of IL-4 treatment. Four proteins that were reproducibly up-regulated by IL-4 treatment in three separate experiments were characterized, including Cytochrome *c* reductase, Pyrophosphatase, ATP synthase δ chain and Vimentin (Table 1). On the contrary, Valosin, Gelsolin, Alpha enolase (α-enolase) and Vinculin were down-regulated by IL-4 (Table 1).

**Table 1:** Differentially expressed proteins of mature 3T3-L1 adipocytes by IL-4 treatment

Protein identified^a^	M.W. (kD)	Spot no. in Fig. 2	Database accession no.
Up-regulated			
Cytochrome C reductase	53.4	1	BAB28666
Pyrophosphatase	33.1	3	Q9D819
ATP synthase D chain	18.6	4	ATPD MOUSE
Vimentin	53.7	8	CAA39807
Down-regulated			
Vinculin	117	2	AAH08520
Valosin	80.6	5	Q8BNF8
Gelsolin	80	6	Q6PAC1
Alpha enolase	47.3	7	ENOA MOUSE

^a^Each score of above proteins is >62, and protein scores greater than 61 are significant (*p* < 0.05)

Among the IL-4 up-regulated proteins, genotypes of mitochondrial *ATP synthase* subunit are reported to be associated with T2DM pathogenesis (Guo et al. 2005). DeLany *et al.* reveals that induction of ATP synthase δ chain is accompanied by adipogenesis of adipose-derived adult stem cells for promoting glycolysis and fatty acid metabolism (DeLany et al. 2005). Accordingly, the up-regulation of ATP synthase δ chain by IL-4 might result in increased ATP synthesis which promotes adipocyte metabolism. Activity of Pyrophosphatase is to hydrolyze pyrophosphate and produce phosphates which in turn participate in cellular activities such as DNA synthesis, ATP production and signal transduction (Ishibashi et al. 2005). The increased levels of Pyrophospatase under IL-4 treatment also support the above suggestion that IL-4 might facilitate lipid metabolism by promoting ATP production. Cytochrome *c* reductase, a component of complex I in mitochondrial electron transport chain, mediates ATP production and proton release for energy supply (Esteitie et al. 2005). The increased levels of Cytochrome *c* reductase further implicate that efficiency of ATP production may be increased in adipocytes in response to IL-4. Vimentin is one of the important components of cytoskeleton involved in steroid synthesis (Azumi and Battifora 1987). Phosphorylation of Vimentin induces alterations of cytoskeleton which promotes the interaction between lipid droplets and mitochondria, and subsequently steroid synthesis. Moreover, Vimentin is identified in intracellular GLUT4-enriched membranes, suggesting its involvement in glucose transport (Guilherme et al. 2000). Thus, the IL-4-induced Vimentin might contribute to glucose transporter trafficking and glucose uptake (Bluher et al. 2002). Combining the above effects, IL-4 treatment seems to promote adipocyte metabolism by increasing cellular ATP levels.

On the contrary, levels of several proteins are decreased by IL-4 treatment. Valosin is a member of ATPase protein family (Yamamoto et al. 2004). Therefore, IL-4 might elevate intracellular ATP levels by down-regulating Valosin expression. Gelsolin is a Ca^2+^- and polyphosphoinositide- modulated actin-binding protein which serves complicated physiological functions, including regulation of lipid metabolism (Yin et al. 1988). Interestingly, the functions of Gelsolin are in turn regulated by various signaling molecules, including a variety of lipids (Isenberg and Goldmann 1995). Vinculin is another cytoskeleton-associated protein that functions in regulating cell adhesion and motility by transducing force across cell membranes (DeMali et al. 2002; Mere et al. 2005). The IL-4-downregulated Gelsolin and Vinculin might be involved in the regulation of cytoskeleton and secretory proteins. The ubiquitous glycolytic enzyme α-enolase catalyzes the formation of phosphoenolpyruvate from 2-phosphoglycerate, one of the high-energy intermediates that generate ATP in glycolysis (Pancholi 2001). Thus, α-enolase is an enzyme with the functions of regulating glycolysis and cell growth (Saulot et al. 2002). The expression of α-enolase is decreased by IL-4, which may result in decreased glycolysis and pyruvate synthesis, and thus mediate glucose metabolism in adipocytes.

Taking the above observations together, most of the IL-4 regulated proteins are related to or involved in energy metabolism and cytoskeleton dynamics. It suggests that the net effect of IL-4 treatment is to potentiate the increase of ATP levels in adipocytes. The results support our previous observation that adipocytes metabolism might be deviated to catabolism by IL-4 with a favorable condition for lipolysis and inhibiting adipogenesis (Tsao et al. 2014). The catabolized lipids in adipocytes triggered by IL-4 might either be released into periphery or metabolized intracellularlly, and subsequently modulate systemic energy metabolism. However, the underlying mechanism of IL-4 regulating lipid metabolism awaits further investigation. Despite the needs of further study, the protein alterations in adipocytes under IL-4 treatment revealed in the present study provide novel clues into the nature of the interactions between immunological mediators and adipocyte metabolism.

## Conclusion

We previously reported significant associations between *interleukin-4 (IL-4)/IL-4 receptor* genotypes and T2DM, as well as *IL-4* genotypes and HDL-C, which suggest the involvement of IL-4 in lipid metabolism. For further elucidating the role of IL-4 in regulating metabolism, the present study aimed at characterizing the proteins regulated by IL-4 in 3T3-L1 adipocytes. Our results revealed that ATP synthase δ chain, Cytochrome *c* reductase, Pyrophsphatase and Vimentin were up-regulated, whereas α-enolase, Gelsolin, Vinculin and Valosin were down-regulated by IL-4 treatment. IL-4 tends to increase intracellular ATP levels and facilitate energy catabolism in adipocytes by enhancing protein machinery which accelerates ATP synthesis. The results support our previous inference which suggests the metabolism of adipocytes is deviated to catabolism by IL-4 with a favorable condition for lipolysis.

## Materials and methods

### Cell culture and interleukin-4 treatment of 3T3-L1 adipocytes

3T3-L1 pre-adipocytes were maintained in DMEM containing 10 % calf serum in an atmosphere of 5 % CO_2_ at 37 °C. Differentiation of post-confluent cells was initiated by incubation with 0.25 mM dexamethasone, 0.5 mM 3-isobutyl-1-methylxanthine and 10 *u*g/mL insulin for 48 h (Tsao et al. 2014; Hua et al. 2004). Then cells were cultured in DMEM supplemented with 10 % fetal bovine serum and insulin for the next 4–10 days to fully differentiate. Mature 3T3-L1 adipocytes were treated with 50 *n*g/mL recombinant IL-4 (Millipore) for 30 min after 2 h of serum starvation, then cell lysates were harvested and the protein expression profiles were examined as described below.

### Oil Red O staining

Lipid accumulation during 3T3-L1 differentiation was confirmed by Oil Red O staining. Cells were washed twice with phosphate buffered saline, fixed in 10 % formalin neutral buffered solution for 10 min at room temperature, rinsed with distilled water and dried after they were induced. Then cells were stained with Oil Red O (composed of 0.6 % Oil Red O dye dissolved in isopropanol and water, 6:4) for 30 min and washed with distilled water (Tsao et al. 2014; Madsen et al. 2003). The results showed that over 90 % of cells exhibited typical mature adipocytes morphology after 8 days of induction.

### Proteomic techniques

After cells were treated with IL-4, cell lysates were obtained with lysis buffer containing protease inhibitors (8 M urea, 4 % 3-[(3-cholamidopropyl)dimethylammonio]-1-propanesulfonate [CHAPS], 1 mM phenylmethylsulfonyl fluoride [PMSF] and 100 *u*g/*u*L leupeptin) for 30 min on ice, then sonicated by 10 s interval for 3 times. The sonicated cell lysates were frozen at −20 °C for 30 min, and dried out using speed vacuum. The dried cell lysates were re-solubilized in lysis buffer, centrifuged with 11,000 rpm for 30 min at 18 °C. Then supernatant were collected and the proteins in cell lysates were separated by isoelectric focusing (with pH range 4–7) and 9 ~ 15 % gradient SDS-PAGE electrophoresis sequentially. The 2-dimensional electrophoresis gel containing separated proteins was visualized by sliver staining, then the protein spots that showed great alterations before and after IL-4 treatment were cut and washed by sterile distilled water, followed by 50 mM NH4HCO3/100 % acetone nitrite (v/v: 3:2), and de-stained with 0.1 g K3Fe(CN)6 plus sodium thiosulfate solubilized in 50 ml distilled water. The cut gel particles were washed by 25 mM NH4HCO3 and 100 % acetonitrite for dehydration, and soaked in trypsin overnight for in gel digestion, then each sample was applied on the anchor chip for MALDI-TOF analysis.

### MALDI-TOF analysis

MALDI-TOF analysis was conducted as previously described (Wang et al. 2002). Briefly, tryptic peptide solutions were mixed with an equal amount of CHCA matrix (10 mg/mL in 60 % cetonitrite/0.3 % trifluoroacetic acid), spotted onto the sample plates and air-dried. Reflectron mass spectrometric analyses were performed in the Proteomics Core Laboratory in Chang Gung University. Acquired spectra were searched against MASCOT databases. A positive score was defined to be greater than 62 for each peptide ion. Proteins were identified based on multiple matches to peptides from the same protein by MASCOT score.

## Abbreviations

α-enolase: Alpha enolase
CHAPS: 3-[(3-cholamidopropyl)dimethylammonio]-1-propanesulfonate
HDL-C: High density lipoprotein-cholesterol
HSL: Hormone sensitive lipase
IL-4: Interleukin-4
MS: Metabolic syndrome
PMSF: Phenylmethylsulfonyl fluoride
T2DM: Type 2 diabetes mellitus
Th2: Type 2 T helper cell
